# Optimization of N-hydroxysuccinimide ester coupling with aminoallyl-modified RNA for fluorescent labeling

**DOI:** 10.1080/21655979.2020.1765487

**Published:** 2020-05-24

**Authors:** Mengyang Li

**Affiliations:** State Key Laboratory of Microbial Metabolism, School of Life Science and Biotechnology, Shanghai Jiao Tong University, Shanghai, P. R. China

**Keywords:** Fluorescent labeling, N-hydroxysuccinimide, NHS-linked fluorophore, RNA labeling

## Abstract

Site-specific fluorescent labeling of RNA is crucial for obtaining the structural and dynamic information of RNAs by fluorescence techniques. Post-synthetic modification of RNA based on N-hydroxysuccinimide (NHS) coupling reaction is an economic, efficient and simple strategy to introduce fluorophore to samples. However, this strategy are not that frequently used in RNA molecules, and the reported reaction conditions and yields varied among different systems. This study results mainly focused on screening the reaction conditions (reactants concentrations, dimethylsulfoxide concentration, solution conditions, pH and reaction time) between NHS-linked fluorophore and aminoallyl-RNA (aa-RNA) to optimize the yield of fluorescent RNA up to 55%, doubled the initial yield. What’s more, as low as one tenth of fluorescent reagent was used in our protocol compared with the reported protocols, greatly reducing the experimental cost. The protocol can be applied as a general guide potentially for RNA labeling by NHS-ester coupling reaction.

## Graphical Abstract

**Figure:**
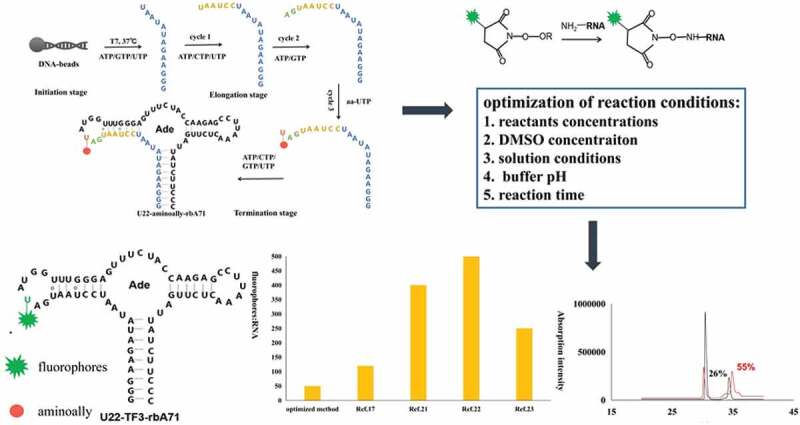

## 1. Introduction

Fluorescent labeling is pivotal to obtain the information of structure and dynamics of RNAs by using fluorescent techniques, such as fluorescent titration, FRET (Fluorescence Resonance of Energy Transfer) and stopped-flow fluorescence [1–5]. Post-synthetic labeling of RNA is applied for preparing fluorescently modified RNAs to alleviate the synthetic bottleneck due to steric inhibition from bulky fluorophores to RNAs [6–8]. And coupling reaction of N-hydroxysuccinimide ester (NHS ester) with primary amino group is a useful strategy for post-synthetic modification of RNAs [5,9,10]. Natural RNAs lack reactive primary amino groups, and site-specific introduction of primary amino to RNAs can trigger the conjugation between NHS ester and the specific site [7]. The urgent requirements of labeling RNAs and ambient experimental conditions bring wide applications of NHS ester coupling reaction in multiple fields, including microarrays, cellular imaging, sample purification, and disease diagnosis [11–17].

**Figure 1.**
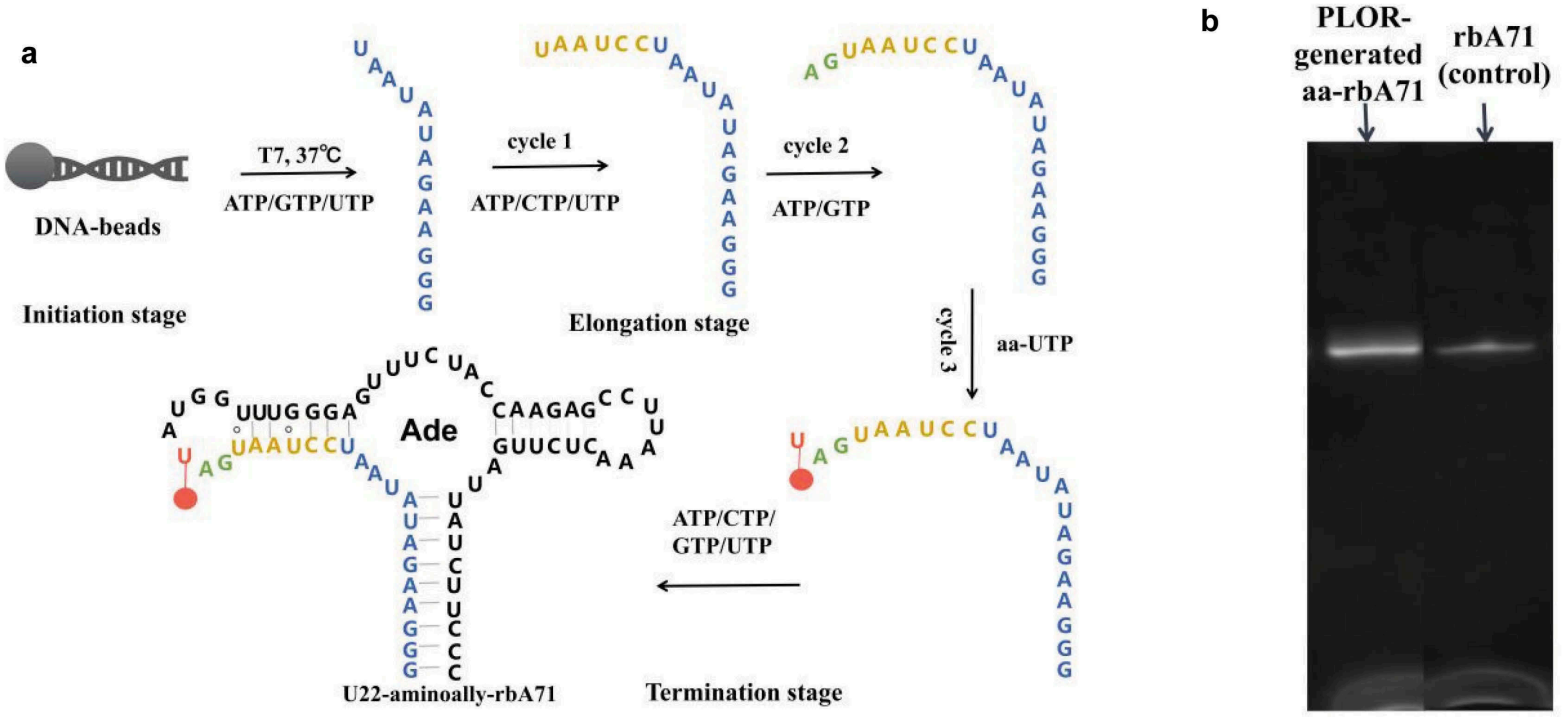
Schematic illustration of incorporation of aminoally group into rbA71 (a) detail procedure for incorporation of 5-aminoally into rbA7. In the initiation, the DNA-beads incubate with T7 RNAP, ATP/GTP/UTP to generate 13nt transcript. T7 RNAP pause at position U13 because of lacking of CTP. In the elongation stage, 3 cycles of elongation stage are performed, and the additional of ATP/CTP/UTP at cycle 1, ATP/GTP at cycle 2 generate a 21nt-transcript;5-aminoally-UTP was added at cycle 3 to incorporate 5-aminoally modification into rbA71. In the termination stage, the addition of ATP/CTP/GTP/UTP completes the transcription of rbA71. (b) Gel visualized for PLOR-generated aminoally-modified RNA and standard rbA71 samples. Loading volume of aa-RNA and rbA71 is 2 µl and 1.5 µl.

Here, 71nt adenine riboswitches aptamer domain (rbA71) was used as a model RNA to optimize the NHS ester coupling reaction for fluorescent labeling of RNAs. rbA71 located at the 5ʹ untranslated region of mRNA, regulates the downstream gene expression by binding with adenine [18–20]. The aminoally-modified rbA71 (aa-rbA71) was synthesized by incorporation of 5-aminoally-UTP into Site 22 of rbA71 (Site 22 is labeled in red in Figure 1(a)) to obtain aa-rbA71 via PLOR (Position-specific Labeling of RNA) (the detailed synthesis is listed in Material and Methods), which is a novel platform for site-specific labeling of RNA [15,21]. aa-rbA71 reacted with NHS ester-linked Tide Fluor 3 (NHS-TF3) to generate fluorescent-labeled rbA71 following the reported condition [7,10–17,22–26]. Various conditions for NHS coupling reaction were reported. The RNA concentrations change from 5 µM to 1 mM and the ratios of RNA to fluorophores shift from 1:100 to 1:1000. Such excessive usage of fluorescent reagent results in economic burden for labs. The volume ratios of dimethylsulfoxide (DMSO) ranges between 10 and 70% (vol/vol). Sodium bicarbonate solution (NaHCO_3_) or phosphate buffer has been optional in fluorescent labeling of aminoallyl-labeled RNAs [6,7].

Studies on protein or peptides labeling indicated that the NHS ester coupling reaction has been influenced by buffer pH greatly, while the buffer pH for RNA conjugation with NHS-reagent narrows in 7.0–9.0 [7,10–17,22–26]. Besides, the reaction time in some protocols may be shortened to drop the potential degradation of RNAs. Therefore, aims of this work was to: (1) screened various conditions, including concentration of reactants, DMSO concentration, solution condition, pH and reaction time to systematically optimize the reaction conditions; (2) explored a new strategy for fluorescent labeling of RNAs, which significantly increase the labeling efficiency, shorten the reaction time and consistency, reduce cost.

## 2. Materials and methods

### 2.1. Synthesis of aminoallyl-labeled RNA by PLOR

3 mL PLOR reaction, containing initiation stage, three elongation cycles, and termination stage was used to incorporate 5-Aminoallyl-UTP (Trilink, N1062) into Site 22 of rbA71. The detailed procedure and reagent usage were performed as the following: In the initiation stage, 5 µM T7 RNA Polymerase (T7 RNAP), 5 µM solid-phase bead-DNA template, 0.48 mM ATP, 0.48 mM GTP and 48 µM UTP were incubated in the initiation buffer (40 mM pH 8.0 Tris-HCl, 100 mM potassium sulfate, 6 mM magnesium sulfate, 10 mM Dithiothreitol, pH 8.0) at 37°C for 15 min to initiate synthesis. The transcription in the initiation was paused due to CTP missing with the production of a 13nt (nucleotides) transcript (shown in blue in Figure 1(a)). The bead-DNA was filtered and rinsed 5 times by 1 mL washing buffer (40 mM pH 8.0 Tris-HCl, 6 mM magnesium sulfate, pH 8.0) to remove the residual NTPs after the initiation cycle. The bead-DNA template used in the PLOR reaction was prepared by immobilized the ordered DNA (Table 1) on the neutravidin-coated agarose beads (SMART, SA021010) as described earlier [15].

**Table 1.** Sequence of DNA template for PLOR.

DNA template	Sequence
Noncoding	5ʹ-TCTGATTCAGCTAGTCCATAATACGACTCACTATAGGGAAGATATAATCCTAATGATATGGTTTGGGAGTTTCTACCAAGAGCCTTAAACTCTTGATTATCTTCCC-3’
Coding	5ʹ-GGGAAGATAATCAAGAGTTTAAGGCTCTTGGTAGAAACTCCCAAACCATATCATTAGGATTATATCTTCCCTATAGTGAGTCGTATTATGGACTAGCTGAATCAGA-3’

In the first elongation cycle, 10 µM ATP (Invitrogen,18330019) 10 µM CTP (Invitrogen, 18331017) and 10 µM UTP (Invitrogen,18333013) were added to the reactor and the incubated for 10 min at 25°C, pausing again due to lack of GTP (Invitrogen, 18332015) and lengthening 6 nt (in yellow at Figure 1(a)) to the transcript. After filtering and rinsing the bead-DNA for 5 times, we added 5 µM ATP and 5 µM GTP to proceed the transcription to site 21 (shown in green, Figure 1(a)). After the routine filter and rinse, the aminoallyl (aa) group was introduced to the transcript at the cycle 3, elongation with the addition of 5 µM aa-UTP and incubation at 25°C for 10 min, extending the transcript to aa-U22 (shown in red at Figure 1(a)), followed by filtration and bead-rinsing. In the termination stage, 55 µM ATP, 55 µM CTP, 45 µM GTP and 90 µM UTP were mixed with the solid-phase complexes at 25°C or 10 min to complete the transcription of full-length U22-aa-rA71. 3.3 nmol of labeled RNA was obtained after purification by 15% (wt/vol) denaturing PAGE. Lyophilize the U22-aa-rbA71 using freeze dryer (2–8 LD-PLUS, Christ Beta, Germany) and stored at −80°C for further experiments.

### 2.2. Post-synthesis fluorescent labeling TF3 by conjugation between NHS-TF3 and aminoallyl-modified RNA

#### 2.2.1. Original labeling methods

1 mg of NHS-TF3 (AAT Bioquest, 2271) was dissolved in 40 µl of DMSO (Molecular Probes, D12345) and divided into aliquots of 2 µl. To 2 µg of aa-modified RNA in 3.33 µl of DEPC-treated water, 1.66 µl of NaHCO_3_ (Macklin, S818360-500 g) buffer (300 mM, pH 9.0) and 5 µl of DMSO was added. After addition of bicarbonate buffer, the aa-RNA was mixed with NHS-TF3. Repeated pipetting incubated reaction solution for 1.5 h at room temperature in the dark.

#### 2.2.2. To optimization of reaction conditions

Take optimization of reactants concentration for example: dissolved the lyophilized aa-RNA (0.3 nmol) in diethylpyrocarbonate-treated (Amresco, E174-100 G) water (DEPC-treated water), mixed with 5.0 µl of DMSO and 1.0 µl of sodium bicarbonate buffer (300 mM, pH 9.0). The resulting solution was treated with 0.6 µl of 5 mM NHS-TF3, 0.5 µl, 1 µl of 30 mM NHS-TF3, 0.4 µl, 1 µl of 150 mM NHS-TF3 dissolved in DMSO respectively, additional DEPC-treated water was added to take the total reaction volume to 10 µl. Vortex, following incubation at 28°C in the dark for 4 h. Then kept NHS-TF3 at 15 mM (added to 1 µl of 150 mM NHS-TF3 dissolved in DMSO), aa-RNA concentrations were increased to 0.1 and 0.3 mM (1 and 3 nmol aa-RNA dissolved in DEPC-treated water, respectively). As for optimization of DMSO, solution conditions and reaction times, concentration of aa-RNA and NHS-TF3 was kept at 30 µM and 1.5 mM. For 10 µl of reaction volume, 10% (vol/vol) is 1 µl and so on. In each optimization experiment, another reaction conditions and the detail procedure were same as the protocol described in optimization of reactants concentration while change the reaction condition needing optimized.

#### 2.2.3. Refined labeling methods

One vial of mono-reactive dyes (1 mg) was dissolved in 10 µl of DMSO and divided into aliquots of 1 µl. To 3 nmol of aa-modified RNA dissolved in DEPC-treated water, 5.5 µl of DMSO and 1.0 µl of 300 mM sodium bicarbonate buffer with pH 7.0 was added. The resulting solution mixed with 1 µl of NHS-TF3 (150 mM) dissolved in DMSO. Additional DEPC-treated water was added to take the total reaction volume to 10 µl. Vortexed well, the following incubation at 28°C in the dark for 30 min.

### 2.3. Detection of reaction efficiency

The yields of reaction conditions optimizations were detected by denaturing (10 M Urea) polyacrylamide gels (typically 15% and 1.5 mm thickness). Samples were diluted in 3 volume of loading buffer (1x TBE, 90% urea) prior to gel loading. 1.5 mm gels were generally run at 200 V. Fluorescent labels were directly visualized on a scanner (9400, Typhoon, US). The refined and original reaction efficiency was determined by High-Performance Liquid Chromatography (HPLC) (LC-20A, SHIMADZU, Japan) at absorption spectrum of 260 nm. Run the sample on the following HPLC program: 10 min at 10% buffer B (90% buffer A) and then ramp from 10 to 50% buffer B over 50 min, ramp from 50 to 80% over 70 min (flow rates are 0.5 ml/min).

## 3. Results

### 3.1. Incorporation of 5-aminoallyl-modified nts into RNA

According to the mechanism of PLOR [14], 6 step PLOR was performed to obtain the aminoally-modified rbA71 (U22-aa-rbA71), the detail protocol is listed in method and materials. As shown in Figure 1. T7 RNAP, DNA-beads and ATP/GTP/UTP was incubated for 10 min at 37°C in the initiation stage, producing first 13nt transcript. Different NTP(s) group were added to each cycle of elongation stage until 5-aminoallyl-modified UTP was incorporated into RNA at cycle 3 of elongation stage. ATP/CTP/GTP/UTP were added to complete the transcription of U22-aa-rbA71. U22-aa-rbA71 was loaded at denaturing 15% (wt/vol) polyacrylamide gel, migrating similar as its unmodified counterpart (Figure 1(b)), and the yield for U22-aa-rbA71 generated from PLOR is averaged to be 30.0%.

### 3.2. Optimization of reaction conditions

**Figure 2.**
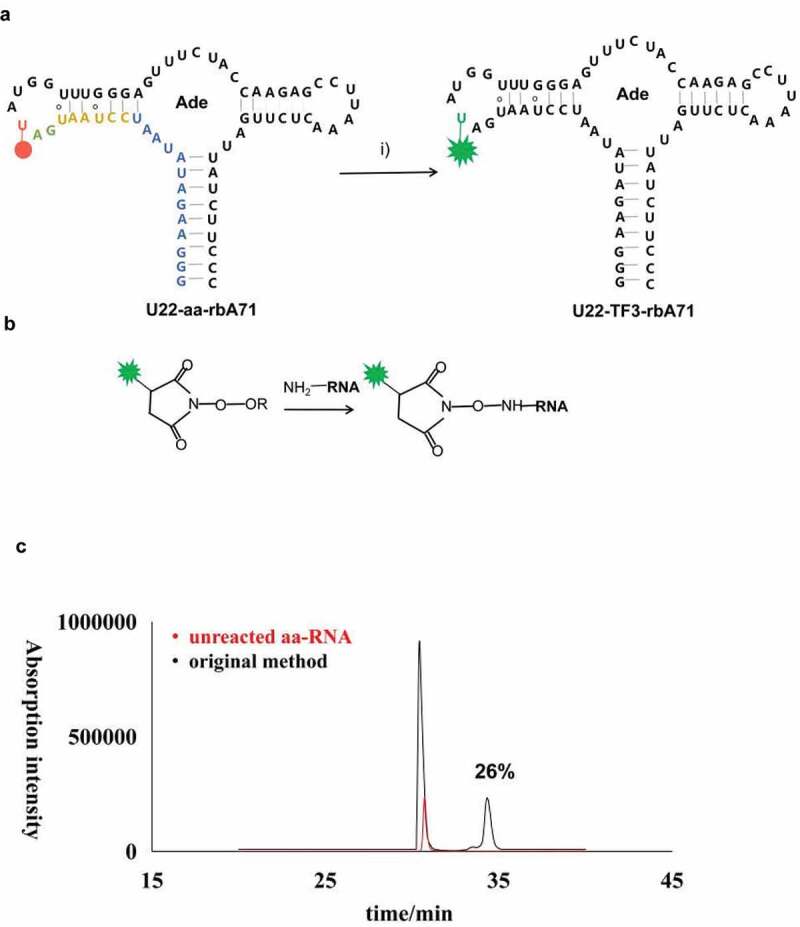
The schematic procedures of coupling reaction of aa-RNA and NHS ester-linked TF3; (a) Schematic illustration of the labeling workflow at U22 position. Residues highlighted on secondary structure of U22-aa-rbA in blue represent the first 13 nucleotides synthesized in the initiation stage, residues highlighted in orange, gree, and red represent 3step in elongation stage. The aminoally atom is represented by red dot at position 22, and TF3 is represented by green star. (b) Illustration of reaction between aminoallyl-modified RNA and NHS ester-linked fluorescent dyes. (c) HPLC profiles for original method efficiency. The spectra in black is original method, in red is unreacted aa-RNA.

**Figure 3.**
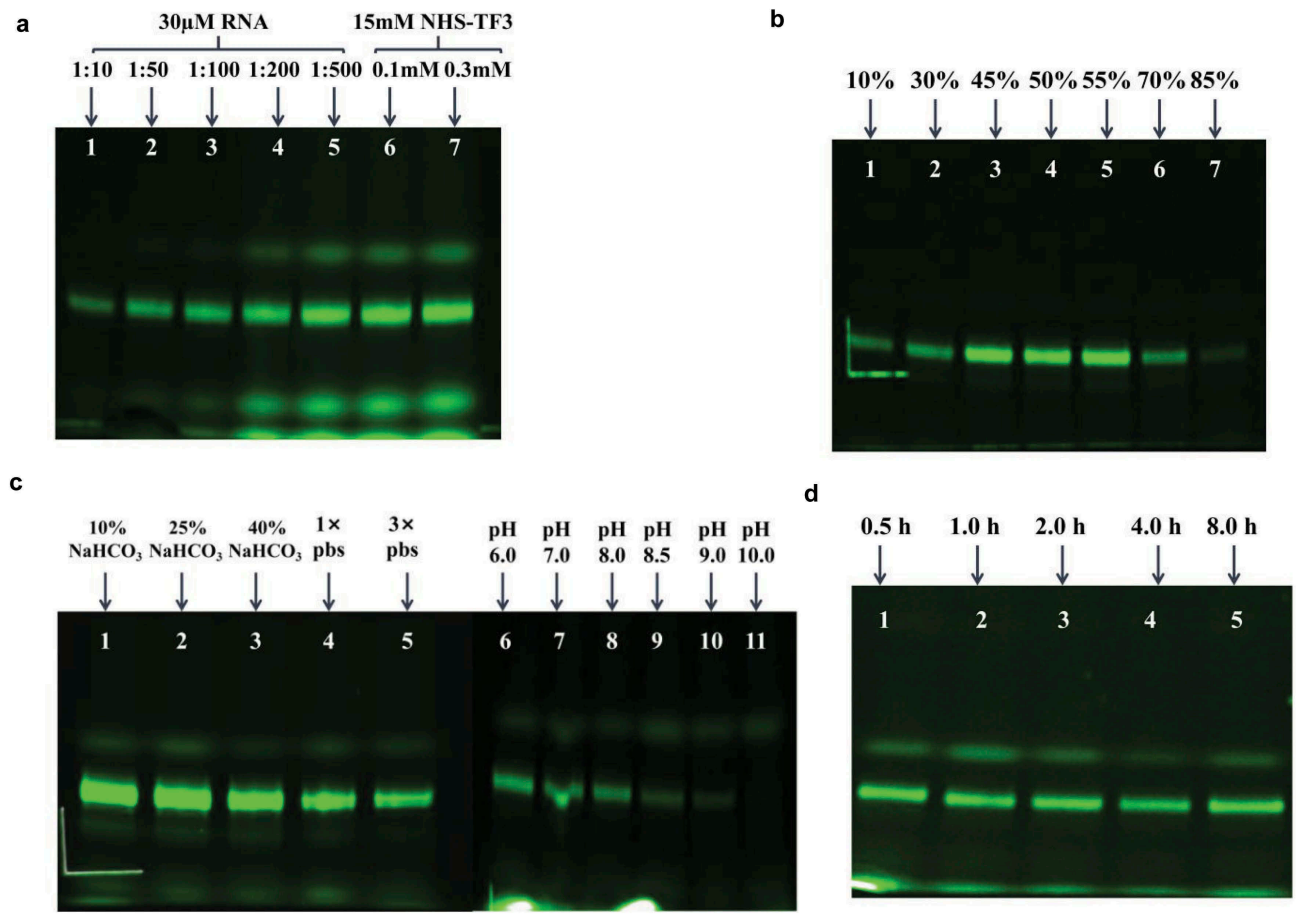
Optimization of reaction conditions (reactants concentrations, dimethylsulfoxide concentraion, solution conditions, pH and reaction time) for fluorescent labeling of aa-rbA71. (a) Gel images of crude products at increasing RNA to NHS-TF3 ratio while keeping the concentration of aa-RNA at 30 µM: 1:10of RNA to NHS-TF3 ratio (lane 1), 1:50 of RNA to NHS-TF3 ratio (lane 2), 1:100of RNA to NHS-TF3 ratio (lane 3), 1:200of RNA to NHS-TF3 ratio (lane 4) and 1:500of RNA to NHS-TF3 ratio (lane 5). Keep the NHS-TF3 concentration at 15 mM, increase the concentration of aa-RNA to 0.1 mM (lane 6) and 0.3 mM (lane 7). (b) Gel images of fluorescent products at various concentration of DMSO: 10% (vol/vol) of DMSO (lane 1), 30% (vol/vol) of DMSO (lane 2), 45% (vol/vol) of DMSO (lane 3), 50% (vol/vol) of DMSO (lane 4), 55% (vol/vol) of DMSO (lane 5), 70% (vol/vol) of DMSO (lane 6) and 85% (vol/vol) of DMSO (lane 7). (c) Gel visualized for fluorescent products at different solution conditions: 10% NaHCO_3_ buffer (0.3 M, pH 9.0) (lane 1), 25% NaHCO_3_ buffer (0.3 M, pH 9.0) (lane 2), 40% NaHCO_3_ buffer (0.3 M, pH 9.0) (lane 3), 1× pbs buffer (lane 4), and 3× pbs buffer (lane 5); Change 10% 0.3 M NaHCO_3_ buffer PH, gel image of fluorescent products at pH 6.0 (lane 6), pH 7.0 (lane 7), pH 8.0 (lane 8), pH 8.5 (lane 9), 9.0 (lane 10) and 10.0 (lane 11). (d) Gel visualized for fluorescent products at different reaction time: 0.5 h (lane 1), 1 h (lane 2), 2 h (lane 3), 4 h (lane 4) and 8 h (lane 5).

In my hand, the labeling efficiency of TF3 to U22-aa-rbA71 is about 26.0% (Figure 2) following the reported protocol as described in method and materials [14].This research optimized five factors that may affect the conjugation reaction to improve the labeling yields for rbA71. Based on the reported protocol described above, change the ratio between NHS-TF3 and aa-rbA71 from 10:1 to 500:1 (aa-rbA71 was kept at 30 µM), it is found that the labeling efficiency was significantly improved as more NHS-TF3 (Figure 3(a)), and 2.5 more TF3-rbA71 was detected at 500:1 than at 10:1 (Figure 3(a)). Keep the concentration of fluorophores at 15 mM and higher concentrations of aa-RNA, 100, 300 µM aa-RNA were tested. The yield of aa-RNA labeling reaction has been slightly increased as aa-rbA71 reached 0.3 mM, and NHS-TF3 was 15 mM.

As DMSO increased from 10 to 45% (vol/vol), the labeling efficiency was obviously improved (Figure 3(b)), however, no further improvements were observed as DMSO occupied 55% in the reaction. What’s more, the labeling efficiency dropped significantly as DMSO reached 70%, and no fluorescent products were detected. Labeling efficiency is also sensitive to the buffers. Keep reaction condition under 30 µM aa-RNA, 1.5 mM NHS-TF3, 55% (vol/vol) DMSO,10, 25, and 40% (vol/vol) of NaHCO_3_ (300 mM, pH 9.0) and 1×, 3× PBS buffer (pH 7.4) were screened for the fluorescent strategy (Figure 3(c)). Higher yields were observed with NaHCO_3_ buffer (0.3 M) than PBS buffer. No obvious changes in yields were observed from 10 to 25% NaHCO_3_, however, about 50% less labeled sample was obtained at 40% NaHCO_3_ (Figure 3(a)). In addition, the production of TF3-rbA71 changed with pH 7.0–10.0 at 25% NaHCO_3_. The yields were optimized at 7.0–7.5. Less products were detected at higher pH until barely no products were obtained at pH 10.0, which is different from the protocols for protein labeling. (Figure 3(c)). To optimize reaction time, the reactions were ran at 0.5, 1, 2, 4, and 8 h, surprisingly, the reactions were insensitive to reaction time (Figure 3(d)). To avoid the potential of RNA degradation, I chose 0.5 h to be the optimal reaction time.

## 4. Discussion

**Figure 4.**
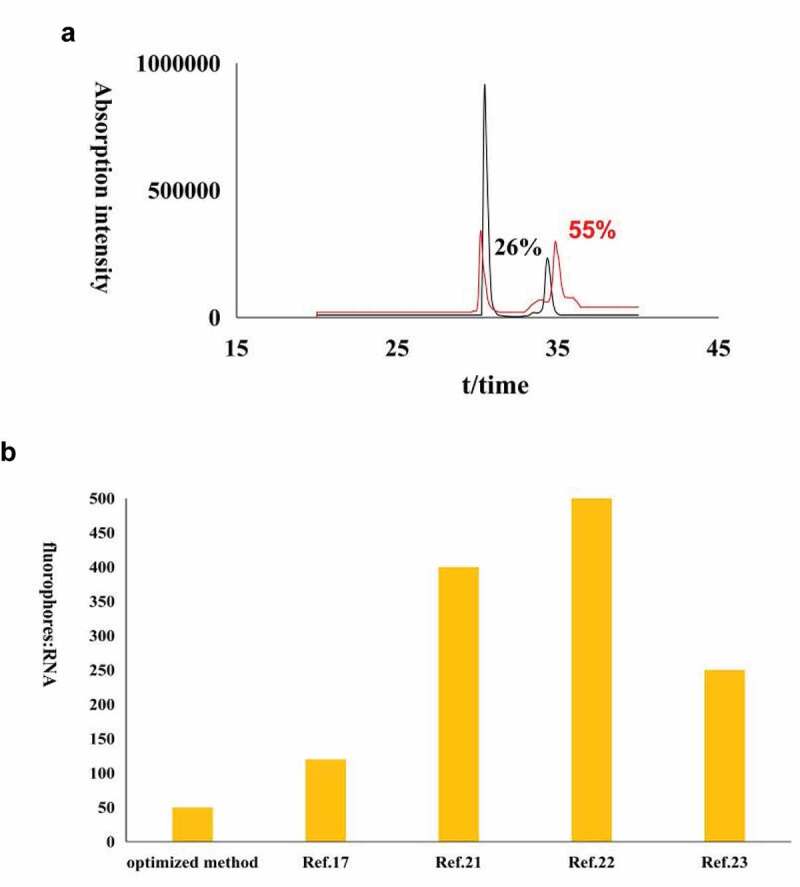
High-Performance Liquid Chromatography profiles for the optimized method and original method; (a) High-Performance Liquid Chromatography spectra of optimized method and original method and (b) Comparison for RNA to dye ratio between optimized method in present study and reported methods [14–16,24].

With stronger research interest in structural and dynamics of RNAs, fluorescent RNAs are in great request. However, preparation of fluorescent-labeled samples was inhibited to some extent by high cost of fluorescent reagents or complicated experimental procedures. Coupling between NHS ester and aminoallyl offers a convenient way to introduce fluorophores to RNAs as multiple fluorophore-NHS and aminoallyl-NTP are commercial. We optimized the labeling efficiency of the labeling strategy by screening various conditions. Based on my results: when 0.3 mM aa-RNA mixed with 15 mM NHS-TF3 (RNA to dye ratio is 1:50), incubated with 55% (vol/vol) DMSO and 10% (vol/vol) NaHCO_3_ buffer (300 mM, pH 7.0) for 0.5 h at 28°C in the dark, the degree of fluorescent labeling is 55%, which is two times higher than the reported protocols [14], and the cost of fluorophores is 2–10 times less than published protocols [11–17,22–26] (Figure 4).

Growing degree of aa-RNA fluorescent labeling observed in higher concentration of aa-RNA at the same RNA to dye ratio. This implies that higher concentration of aa-RNA may more efficiently couple to NHS-ester-linked dye thus decrease the ratio of RNA to fluorophores. However, restricting by experimental cost, the concentrations of aa-RNA was just increased to 0.3 mM, higher concentration of aa-RNA are suggested for users in future studies, which may further enhance the reaction efficiency and reduce the cost. Unlike most of protocols for DNA or protein labeling [24,27,28], this work demonstrated that the optimal concentration of DMSO is 45–55% (vol/vol). Suggesting that sufficient DMSO was required to achieve efficient labeling of aa-RNA, and the concentration of DMSO should be kept at 45–55% (vol/vol) while avoiding high (>70%) or low (<30%) concentration of DMSO. NaHCO_3_ (300 mM) buffer has been indicated that is more suitable for fluorescent labeling of aa-RNA than PBS buffer, the optimal buffer pH in this study is 7.0–7.5. Thus, 10–25% (vol/vol) NaHCO_3_ buffer (pH 7.0–7.5, 300 mM) is recommended as reaction buffer for users. Shortening the reaction time to 0.5 h is another advantage of present conditions. Such short experimental time is favorable for RNAs as most RNAs are fragile for degradation. Although the RNAs tested in this research are limited and the actual relationship between yields and reaction conditions may be much more complicated than exposed in this work, present finding can be applied as a general guide potentially for RNA labeling by NHS-ester coupling reaction to facilitate fluorescent studies.

## 5. Conclusions

In conclusion, the results indicated the effects of various reaction conditions (the concentrations of reactants, pH, and reaction time) on fluorescent labeling of RNAs by using NHS and aminoallyl coupling reaction. The labeling efficiency increased 2 times by using optimized conditions with the use less fluorophore-labeled NHS compared with the reported protocol. Briefly speaking, present protocol reached higher efficiency with less cost and time.

## Highlights

1. Systematically optimize the reaction conditions of coupling reaction for fluorescent labeling of aminoally-modified RNA.
2. Strongly Increase the efficiency of fluorescent labeling of aminoally-modified RNA
3. Significantly reduce the cost of fluorescent labeling of RNA via decrease the RNA to dye ratio.
4. Shorten the reaction time for fluorescent labeling of aminoally-modified RNA
